# The Emerging Roles and Therapeutic Potential of Soluble TREM2 in Alzheimer’s Disease

**DOI:** 10.3389/fnagi.2019.00328

**Published:** 2019-11-26

**Authors:** Li Zhong, Xiao-Fen Chen

**Affiliations:** ^1^Fujian Provincial Key Laboratory of Neurodegenerative Disease and Aging Research, Institute of Neuroscience, Medical College, Xiamen University, Xiamen, China; ^2^Shenzhen Research Institute of Xiamen University, Shenzhen, China

**Keywords:** Alzheimer’s disease, microglia, soluble TREM2, biomarker, neuroinflammation

## Abstract

Alzheimer’s disease (AD) is the most common form of dementia characterized by the deposition of extracellular amyloid-β (Aβ)-containing plaques, the formation of intraneuronal neurofibrillary tangles as well as neuroinflammatory changes. As the key player in the brain innate immune system, microglia has now taken a center stage in AD research. A large number of AD risk loci identified by genome-wide association studies are located in or near the genes highly expressed in microglia. Among them, the triggering receptor expressed on myeloid cells 2 (TREM2) has drawn much attention. A rare variant in TREM2 increases AD risk with an odds ratio comparable to the strongest genetic risk factor apolipoprotein ε4 allele. In the past 6 years, extensive studies have dissected the mechanisms by which TREM2 and its variants modulate microglial functions impacting amyloid and tau pathologies in both animal models and human studies. In addition to the full-length TREM2, research on the soluble form of TREM2 (sTREM2) has facilitated the translation of preclinical findings on TREM2. In this review, we summarize our current understanding of the biology and pathobiology of sTREM2 including its origin, its emergence as a disease biomarker, and its potential neuroprotective functions. These aspects are important for understanding the involvement of sTREM2 in AD pathogenesis and may provide novel insights into applying sTREM2 for AD diagnosis and therapy.

## Introduction

Alzheimer’s disease (AD) is the most common cause of dementia in the elderly, typically presenting with a slow but progressive behavioral and cognitive impairment. Key histopathological hallmarks of AD include the deposition of extracellular neurotoxic plaques primarily composed of Aβ, and intracellular neurofibrillary tangles resulting from the aggregation of hyperphosphorylated tau (Holtzman et al., 2011). In addition to protein aggregation, neuroinflammatory changes have emerged as the third core feature of AD pathologies (Heneka et al., 2015; Karch and Goate, 2015). In the central nervous system (CNS), microglia are the resident immune cells that play central roles in neuroinflammation. Recent genome-wide association studies (GWAS) have uncovered a large number of mutations in microglial genes that are highly associated with increased AD risk (Gjoneska et al., 2015; Malik et al., 2015; Efthymiou and Goate, 2017). Hence, it has been proposed that microglia play a much larger role in AD pathogenesis than previously thought. Studies on the new genetic risk factors have aided the rapid progress towards unraveling the multiple facets of microglia under both healthy and diseased conditions (Colonna and Butovsky, 2017; Butovsky and Weiner, 2018; Hammond et al., 2018; Hickman et al., 2018; Heneka, 2019).

During development, microglia are actively involved in eliminating the apoptotic neurons *via* phagocytosis and the refinement of neural circuits by synaptic pruning (Wakselman et al., 2008; Paolicelli et al., 2011; Schafer et al., 2012; Cunningham et al., 2013). In the healthy adult brain, microglial processes are highly motile and constantly survey the surrounding environment in the parenchyma to maintain tissue homeostasis (Davalos et al., 2005; Nimmerjahn et al., 2005). In response to harmful stimuli such as Aβ aggregation, microglia rapidly transform from ramified to amoeboid morphology, facilitating the phagocytosis and clearance of Aβ aggregates (Itagaki et al., 1989; Bolmont et al., 2008). They also proliferate and migrate to the vicinity of plaques, forming a protective barrier around amyloid deposits to reduce the neurotoxicity of amyloid fibrils (Condello et al., 2015; Zhao et al., 2017). However, there is also abundant evidence that microglia have harmful actions in AD. Once activated, microglia can mediate the engulfment of neuronal synapses likely *via* a complement-dependent mechanism. They can also exacerbate tau pathology and secrete detrimental inflammatory factors that can directly or indirectly injure neurons (Hansen et al., 2018).

Hence, microglia may act as a double-edged sword being either protective or detrimental depending on the disease stage. Future efforts in profiling the microglial transcriptome particularly at the single-cell level and correlating such changes with disease progression are necessary to help us better understand the role of microglia in AD pathology (Keren-Shaul et al., 2017; Rangaraju et al., 2018; Hammond et al., 2019). Furthermore, expanding the studies from mouse models to human patients by using human microglia isolated from fresh postmortem brain tissues or human microglia-like cells differentiated from human induced pluripotent stem cells will significantly and greatly increase the success in translational research (Abud et al., 2017; Mizee et al., 2017; McQuade et al., 2018).

Among the AD risk-associated microglial genes, a special interest has been directed at the triggering receptor expressed on myeloid cells 2 (TREM2) since the rare R47H variant of TREM2 increases AD risk almost three-fold (Guerreiro et al., 2013; Jonsson et al., 2013). Thus, the effect size of TREM2 R47H is comparable to that for the ε4 allele of the gene encoding apolipoprotein E (apoE), the strongest genetic risk factor for sporadic AD identified 30 years earlier. As a receptor expressed on microglial cell surface, the ectodomain of TREM2 binds to an array of molecules that are important for AD, including the anionic and zwitterionic lipids, lipoproteins and apolipoproteins, oligomeric Aβ and galectin-3 as reported recently (Atagi et al., 2015; Bailey et al., 2015; Wang et al., 2015; Yeh et al., 2016; Lessard et al., 2018; Zhao et al., 2018; Zhong et al., 2018; Boza-Serrano et al., 2019). While the identities of these ligands remain uncertain, several functions of TREM2 have been well characterized in microglia. Recent studies have suggested that TREM2 impacts a multitude of microglial functions including activation, inflammation, phagocytosis, proliferation, survival and metabolism (Kleinberger et al., 2014, 2017; Cantoni et al., 2015; Wang et al., 2015; Zhong et al., 2015; Yeh et al., 2016; Ulland et al., 2017; Zheng et al., 2017). In the context of AD, TREM2 regulates the recruitment of microglia to the vicinity of amyloid plaque and limits amyloid or plaque tau seeding (Yuan et al., 2016; Cheng-Hathaway et al., 2018; Leyns et al., 2019; Parhizkar et al., 2019). TREM2 is also essential for microglia to eliminate supernumerary synapses in the developing brain (Filipello et al., 2018). Other studies have evaluated the impacts of *Trem2* deficiency, *TREM2* mutation, or human TREM2 expression in amyloid or tau mouse models (Ulrich et al., 2014; Jay et al., 2015; Wang et al., 2015; Bemiller et al., 2017; Leyns et al., 2017, 2019; Cheng-Hathaway et al., 2018; Lee et al., 2018; Sayed et al., 2018; Song et al., 2018). Surprisingly, these studies have produced conflicting results, with reports that AD-related pathology could be either increased or decreased in the presence of TREM2. It was proposed later that TREM2 modulates AD pathology dependent on disease progression (Jay et al., 2017; Shi and Holtzman, 2018). Future studies in comprehensive transgenic and knockout animal models are necessary to unravel the complex pathogenic roles for TREM2.

In addition to the full-length TREM2, research on the soluble form of TREM2 (sTREM2) has facilitated the translation of preclinical findings on TREM2. sTREM2 was produced from either the proteolytic cleavage of the membrane-anchored TREM2 receptor or the alternative splicing of TREM2 lacking the transmembrane domain (Schmid et al., 2002; Wunderlich et al., 2013). The levels of sTREM2 in the cerebrospinal fluid (CSF) have been quantified and found to be elevated in AD (Heslegrave et al., 2016; Piccio et al., 2016; Suárez-Calvet et al., 2016a). In cross-sectional studies, the levels of sTREM2 change dynamically during the progression of AD, peaking at the early symptomatic stages of the disease (Suárez-Calvet et al., 2016b). Importantly, the CSF concentrations of sTREM2 positively correlate with the levels of total tau and phosphorylated-tau (p-tau) in the CSF. These observations suggest that sTREM2 may play an important role in the development of AD pathology and neurodegeneration.

The presence of sTREM2 was once regarded as the loss-of-function consequences of full-length TREM2 on the cell surface. It could be either an inactive by-product from full-length TREM2 shedding or a decoy receptor opposing full-length TREM2 signaling. Nevertheless, emerging evidence from our study and others suggests that sTREM2 is actively involved in regulating microglial dynamics and AD-associated pathology. In this review article, we summarize the exciting research progress in the knowledge of sTREM2 including its origin, its biological and pathobiological functions. We further discuss the potential application of sTREM2 in AD diagnosis and therapy.

## The Origin of Soluble TREM2

**Figure 1:**
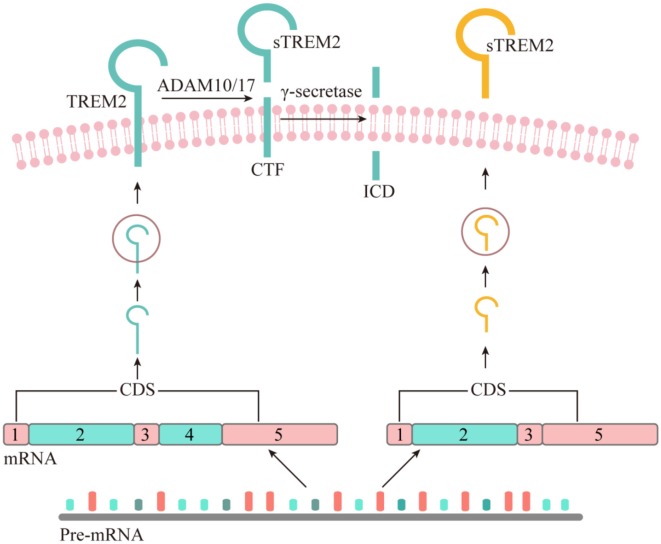
The origin of soluble form of TREM2. Although all the potential physiological sources of sTREM2 remain to be determined, recent studies suggest that sTREM2 can be generated by either alternative splicing or proteolytic cleavage of the full-length TREM2 protein. The *TREM2* gene has three different alternative transcripts. The canonical and also the longest TREM2 transcript consists of five exons, with exon 4 encoding a transmembrane domain. This isoform is anchored to the cell membrane and shed by ADAM10 and/or ADAM17, leading to the production of a soluble TREM2 and a C-terminal fragment (CTF). The TREM2-CTF is further cleaved by γ-secretase to generate a TREM2 intracellular domain (ICD). It remains to be determined whether the second-longest transcript of TREM2, which lacks exon 5, can generate sTREM2 *via* a sequential proteolytic processing. The shortest TREM2 transcript encodes a soluble form of TREM2 due to the lack of exon 4 which encodes the transmembrane domain of the receptor.

The existence of sTREM2 at the protein level was first reported in 2008 by immunoblotting of the precipitates from either human CSF or cultured dendritic cells supernatant fluid (Piccio et al., 2008). The sTREM2 protein was found to carry different levels of glycosylation and its size was reduced to 20 kDa after O- and N-deglycosylation. Interestingly, a TREM2 transcript that lacks the transmembrane domain was described in human and mouse myeloid cells (Figure 1), leading to the hypothesis that sTREM2 originates from alternative splicing of TREM2 (Schmid et al., 2002; Begum et al., 2004; Melchior et al., 2006). A recent study using bulk RNA-Seq data in the human brain tissue further demonstrates that around 25% of the sTREM2 protein originates from the TREM2 transcript that lacks the transmembrane domain (Deming et al., 2019). A strong and significant correlation of the sTREM2 band detected by Western-blots was found with the transcript that codifies for the soluble form of TREM2 (*R*^2^ = 0.42, *p* < 0.05). However, the predicted molecular weight of this transcript is ~27 kDa, which is larger than the 20 kDa deglycosylated sTREM2 protein detected in the CSF. Therefore, alternative splicing may not be the major source of sTREM2 protein.

As a single-pass type I transmembrane protein, TREM2 undergoes sequential proteolytic processing similar to the well-characterized amyloid precursor protein (APP; Wunderlich et al., 2013). The initial cleavage of TREM2 by a shedding enzyme results in the liberation of sTREM2 into the extracellular space. It was later reported that a disintegrin and metalloproteinase domain-containing protein (ADAM) family, including ADAM10 and ADAM17, are the proteases involved in the shedding of TREM2 ectodomain (Kleinberger et al., 2014; Feuerbach et al., 2017; Schlepckow et al., 2017; Thornton et al., 2017). However, which enzyme plays a dominant role remains controversial. In 2017, two independent groups reported simultaneously that sTREM2 is generated through the ADAM10/ADAM17 cleavage at the H157-S158 peptide bond (Schlepckow et al., 2017; Thornton et al., 2017). Interestingly, a late-onset AD-associated H157Y mutation significantly increases the shedding of full-length TREM2 in HEK293 cells, resulting in elevated levels of sTREM2 in the conditioned media.

## The Genetic Modifiers of Soluble TREM2 Levels

The levels of sTREM2 in the CSF were quantified and found to be elevated in patients with AD and other neurological disorders with inflammatory etiologies (Piccio et al., 2008; Heslegrave et al., 2016). However, very little is known with regards to the mechanisms underlying sTREM2 generation under physiological and pathological conditions. Given that TREM2 mutations can affect protein production, maturation and cleavage, the amounts of sTREM2 in the CSF are likely influenced by TREM2 genetic status. Several studies have measured the CSF levels of sTREM2 in individuals carrying different TREM2 genetic variants and found these variants brought varying effects on sTREM2 levels in the CSF (Piccio et al., 2016; Deming et al., 2019). Carriers of the NHD-associated variants Q33X and T66M presented significantly lower CSF sTREM2 levels than controls. Similarly, the T96K/L211P/W191X carriers also had lower sTREM2 levels compared to controls. In contrast, the CSF sTREM2 levels were significantly higher in R47H carriers compared to non-carriers. Therefore, these variants may alter the sTREM2 level and increase the disease risk through distinct pathogenic mechanisms.

To identify more genetic modifiers of CSF sTREM2 levels, Deming et al. (2019) performed a GWAS and found the membrane-spanning 4-domains superfamily A (*MS4A*) gene cluster as a key modulator of sTREM2 concentration in the CSF. Variants in the *MS4A* gene region have been previously linked to the risk of developing LOAD (Hollingworth et al., 2011; Naj et al., 2011; Lambert et al., 2013). Among them, the rs1582763 which increases the expression of both *MS4A4A* and *MS4A6A* genes in the brain is associated with elevated sTREM2 levels in the CSF, reduced AD risk and delayed age at onset. In contrast, the rs6591561 which creates a missense variant within the *MS4A4A* gene (M159V) is associated with reduced CSF sTREM2 levels, increased AD risk and accelerated age at onset. The study further provided functional evidence that CSF sTREM2 levels can be modified *via* modulating *MS4A4A* expression or targeting *MS4A4A* with a specific antibody. However, the study only focused on the common variants with a minor allele frequency larger than 0.02. Therefore, additional studies in a larger sample size will be necessary to fully identify the genetic modifiers of CSF sTREM2 levels. Moreover, future studies are necessary to uncover the molecular mechanisms by which the *MS4A* gene cluster modulates sTREM2 level. The MS4A transmembrane proteins are thought to play a role in intracellular protein trafficking (Cruse et al., 2015). It would be interesting to investigate whether the MS4A family regulates the trafficking of key proteins involved in sTREM2 generation, including the full-length TREM2 and ADAM10/17.

## Soluble TREM2 as a Potential Disease Biomarker

The first attempt to quantify the levels of sTREM2 in the CSF was carried in subjects with multiple sclerosis (MS) and other CNS inflammatory diseases (Piccio et al., 2008). The amounts of sTREM2 were found to be significantly elevated in these subjects as compared to non-inflammatory conditions. In accordance with the finding, another study also observed higher CSF levels of sTREM2 in MS subjects than healthy controls, regardless of their clinical stages (Öhrfelt et al., 2016). Interestingly, after treatment with an anti-inflammatory drug natalizumab, there was a clinical improvement and simultaneously the levels of sTREM2 were decreased to levels comparable to the controls. Therefore, the CSF sTREM2 levels might serve as a biomarker for inflammatory diseases including MS, and it could be used to monitor the effects of anti-inflammatory drugs or the efficacy of therapeutics in the clinic.

Since TREM2 genetic variants are linked to AD, the CSF sTREM2 levels in AD cohorts have been extensively measured and correlated to the pathological changes in AD. By using either mass spectrometry or ELISA assay to quantify the amounts of sTREM2, two independent studies both reported significantly higher levels of sTREM2 in the CSF of mild AD subjects than healthy controls (Heslegrave et al., 2016; Piccio et al., 2016). Importantly, they showed that the CSF sTREM2 levels were positively correlated with that of total tau and p-tau. However, no significant correlation between sTREM2 and Aβ42 levels in the CSF was observed, suggesting that sTREM2 might play functions in the pathological processes occurring after Aβ accumulation. In a cross-sectional study, the CSF levels of sTREM2 were traced during the clinical course of AD, including the preclinical AD, mild cognitive impairment (MCI) due to AD (MCI-AD), and AD dementia (Suárez-Calvet et al., 2016b). Interestingly, the CSF levels of sTREM2 increase in a disease stage-dependent manner, reaching a peak in the early symptomatic phase of AD. The same group further investigated the temporal sequence of changes in CSF sTREM2 levels and markers for amyloid deposition and neurodegeneration as well as cognitive performance (Suárez-Calvet et al., 2016a). They found that the CSF sTREM2 levels were increased before the expected onset of symptoms but after the appearance of amyloidosis and neuronal injury. Thus, the CSF levels of sTREM2 have emerged as a valuable yet dynamic biomarker of the disease progression in AD.

The CSF AD biomarker tests are highly invasive, making them unsuitable for use in the primary care setting. Hence, the sTREM2 levels were also analyzed in the peripheral blood from AD patients as well as healthy subjects. A study has reported higher levels of sTREM2 in the CSF of AD patients, but no significant difference in the plasma levels of sTREM2 between AD cases and healthy controls (Piccio et al., 2016). Similarly, a recent meta-analysis of case-control and AD cohort studies revealed a prominent elevation in the CSF sTREM2 levels in AD groups compared with controls, but no significant difference in the plasma sTREM2 levels between the two groups (Liu et al., 2018). Based on these observations, it appears that only CSF but not plasma levels of sTREM2 could be a promising AD biomarker.

## The Biological Functions of Soluble TREM2

Initially, sTREM2 was postulated to block the function of full-length TREM2 by competing with the ligand binding, in a way similar to the soluble version of another TREM family member, TREM1. Nevertheless, accumulating evidence suggests that sTREM2 possesses important biological and pathobiological functions rather than acting as a decoy receptor opposing full-length TREM2 signaling. One study presented the first evidence that sTREM2 efficiently prevents macrophage apoptosis when cultured under low concentrations of colony stimulating factor 1 (Wu et al., 2015). The study further suggested that sTREM2 promotes cell survival by acting as an intracellular messenger to trigger the activation of extracellular signal-regulated kinases 1/2 and mitogen-activated protein kinase 14. Our studies in microglia also confirmed a protective role of sTREM2 against cellular apoptosis (Zhong et al., 2017). However, sTREM2 forestalled apoptosis *via* activation of the AKT–glycogen synthase kinase 3β–β-catenin pathway in microglia, which is distinct from that in macrophage. Furthermore, sTREM2 induced inflammatory cytokine production in microglia by activating the nuclear factor-κB, accompanied by changes in morphology indicative of microglial activation. These functions of sTREM2 were recapitulated in *Trem2*-deficient microglial cells or mice model, suggesting that sTREM2 acts through a mechanism that is independent of the endogenous, full-length TREM2. Interestingly, sTREM2 derived from the AD-associated variants, R47H and R62H (sTREM2-R47H and sTREM2-R62H) are less potent in both suppressing apoptosis and triggering inflammatory responses in microglia. Thus, our results may constitute previously unknown molecular mechanisms linking TREM2 variants with increased risk of AD.

## The Pathobiological Functions of Soluble TREM2

Since the CSF levels of sTREM2 are intimately associated with AD progression, it has been proposed that sTREM2 might modulate AD pathology. Accumulating evidence from the association analysis between CSF sTREM2 levels and regional brain volumes in AD cohorts suggests that sTREM2 functions are likely neuroprotective. One study reported that higher CSF sTREM2 levels were correlated with increased gray matter volume in MCI patients, when controlled for age, sex, and p-tau-associated brain atrophy (Gispert et al., 2016). A recent study also found that subjects with higher concentrations of CSF sTREM2 showed slower rate of decline in hippocampal volume in early-stage AD (Ewers et al., 2019). Intriguingly, an association between higher CSF sTREM2 concentrations and slower memory decline was observed in individuals with MCI or AD dementia. Furthermore, a higher ratio of sTREM2 to p-tau_181_ levels in the CSF predicted slower rate of clinical progression. These findings suggest that higher CSF sTREM2 levels are associated with attenuated cognitive and clinical decline in AD.

**Figure 2:**
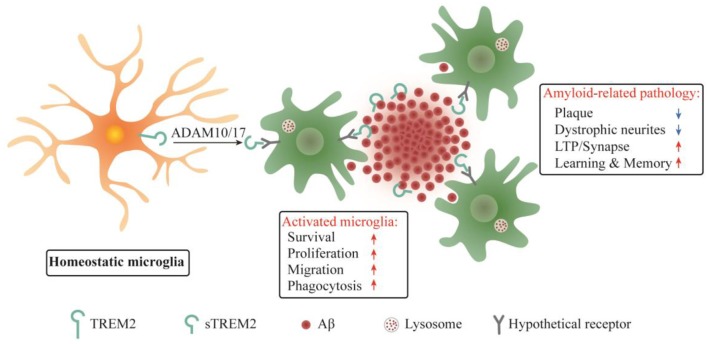
Schematic summary of sTREM2 functions in microglia and amyloid-related pathology. sTREM2 is released into the extracellular space *via* ADAM10 and/or ADAM17 shedding of the membrane-bound TREM2. Upon binding to a hypothetical microglial receptor, sTREM2 converts microglia from a homeostatic to an activated state. It enhances an array of microglial functions, including survival, proliferation, migration, clustering in the vicinity of amyloid plaques, and the uptake and degradation of Aβ. The consequences are the amelioration of amyloid-related pathologies.

It was reported that sTREM2 bound to plaques and neurons when the common variant of human TREM2 was expressed on a bacterial artificial chromosome in *Trem2* deficient 5xFAD mice, while no such findings were observed in mice expressing the R47H variant (Song et al., 2018). Since the R47H mutation constitutes a strong genetic risk factor for AD, the sTREM2 binding to neuron or plaque might have beneficial functions in AD brain. Our recent study in transgenic mouse model of AD provided direct insights into the pathobiological roles of sTREM2 (Zhong et al., 2019). We found that sTREM2 enhances a multitude of microglial functions, including survival, proliferation, migration, clustering in the vicinity of amyloid plaques, and the uptake and degradation of Aβ. Importantly, sTREM2 reduces amyloid plaque load and rescues the functional deficits of spatial memory and long-term potentiation (Figure 2). Depletion of microglia with an inhibitor of colony stimulating factor-1 receptor, PLX3397, abolishes the neuroprotective effects of sTREM2, supporting an indispensable role of microglia in mediating sTREM2 function. Hence, our study demonstrates a protective role of sTREM2 against amyloid pathology and related toxicity likely through modulating microglial activity and raises the interesting possibility that increasing sTREM2 can be explored for AD therapy.

## Future Perspectives

The majority of AD therapies currently under investigation target Aβ accumulation by modulating the activity of key secretases involved in APP processing. Alternatively, antibodies were developed to prevent Aβ aggregation and/or to promote Aβ plaque clearance (Cao et al., 2018). Microglia and related neuroinflammation have been increasingly shown to play key roles in AD pathogenesis. In particular, studies on the functions of TREM2 and its variants in microglia and AD pathogenesis have prompted the design of therapeutic strategies targeting TREM2. While the full-length TREM2 has been suggested to play key roles in AD pathology, results have been conflicting regarding whether TREM2 is beneficial or harmful (Gratuze et al., 2018). In contrast, evidence accumulated from both preclinical and clinical studies suggests a protective effect of sTREM2 against AD pathology. However, the precise mechanisms mediating the functions of sTREM2 remain largely unknown. For example, the microglial surface receptor(s) for sTREM2 and the downstream signaling events remain to be determined. The molecular pathways underlying sTREM2 production also need to be fully elucidated. Since sTREM2 levels vary among different stages of AD, it is important to investigate the impacts of sTREM2 on amyloid and tau pathologies during AD progression. Further studies will be necessary to address these questions and to provide critical mechanistic insights to guide the application of sTREM2 for AD diagnosis and therapy.

## Author Contributions

X-FC and LZ reviewed the literature and drafted the manuscript.

## Conflict of Interest

The authors declare that the research was conducted in the absence of any commercial or financial relationships that could be construed as a potential conflict of interest.
